# Costs of hospital stays in Switzerland during the COVID-19 pandemic: a comparative analysis between cancer and non-cancer patients

**DOI:** 10.1186/s12913-026-14585-0

**Published:** 2026-04-24

**Authors:** Loïc Brunner, Anna Nicolet, Marie-Annick Le Pogam, Isabelle Peytremann-Bridevaux, Jean-Luc Bulliard, Joachim Marti, Karine Moschetti

**Affiliations:** Department of Epidemiology and Health Systems, Unisanté, University Center for Primary Care and Public Health & University of Lausanne, Lausanne, Switzerland

**Keywords:** Cancer, Hospital, Administrative data, Costs, ICU, Covid-19, Lockdown, Comparative time series, Difference-in-differences analysis, Switzerland, ICD-10

## Abstract

**Background:**

The COVID-19 pandemic severely disrupted usual hospital care, leading to widespread cancellations of elective procedures and sharp declines in admissions. Cancer patients, whose treatment depends on timely and coordinated interventions, were particularly vulnerable to pandemic-related disruptions. This study analyzes Swiss hospital cost trends across pre-lockdown, lockdown, and post-lockdown phases to examine differential resource allocation patterns between cancer and non-cancer patients.

**Method:**

A retrospective observational study was conducted using administrative data from 3.9 million inpatient stays among adults. Monthly trends in total hospital stay costs and key cost components, including emergency services, intensive care, operating room, physician, nursing, and imaging services, were analyzed. A difference-in-differences model was employed to assess cost differentials between patient groups across the pandemic periods, adjusting for patient and hospital characteristics.

**Results:**

During the lockdown, the average cost per hospital stay increased by CHF 2,049 (+ 15.7%) and remained CHF 1,654 (+ 12.7%) higher than pre-lockdown levels in the post-lockdown period. Cost increases were especially notable in intensive care unit (CHF + 283) and nursing services (CHF + 759) during the lockdown, with sustained but smaller increases post-lockdown. Once adjusted for patient and hospital characteristics, cancer patients incurred higher hospital costs than non-cancer patients, with the cost differential increasing from 10.0% pre-lockdown to 14.8% during the lockdown and slightly decreasing to 13.8% post-lockdown. This disparity was primarily driven by higher costs of physician services, nursing services, and operating room services. However, ICU costs increased significantly less for cancer patients than for non-cancer patients during lockdown, suggesting differential resource utilization between patient groups.

**Conclusion:**

The COVID-19 pandemic significantly increased average hospital costs in Switzerland, with distinct patterns by patient type and cost category. Despite overall cost pressures, continued attention was maintained for cancer patients, showing resilience in service delivery. Findings underscore the need for cost-informed policy responses to maintain care quality and financial sustainability during health system crises.

**Supplementary Information:**

The online version contains supplementary material available at 10.1186/s12913-026-14585-0.

## Background

The COVID-19 pandemic placed unprecedented strain on hospital systems worldwide. Inpatient services were rapidly restructured to accommodate acute COVID-19 cases, while elective procedures were widely canceled or postponed [1–3]. It is estimated that over 28 million elective surgeries were disrupted globally in the early months of the pandemic [4], with the United States (US) reporting a 48% decrease in total surgical volumes immediately after March 2020 [5]. In most countries, hospital care was disrupted by the pandemic, with a widespread decline in admissions. Considerable variation across settings was observed, with greater reductions among people with less severe illness [6]. In Switzerland, cancellations in elective and emergency procedures also happened and contributed to sharp declines in hospital admissions [7–9]. Concurrently, hospitals around the world faced pressure to expand intensive care unit (ICU) capacity and manage critical workforce shortages, compromising care delivery efficiency and resource allocation [1, 10, 11].

These challenges had significant implications for patients requiring complex, continuous care, most notably individuals with cancer. Delivering care to patients with cancer is inherently complex, requiring timely access to diagnostics, surgical interventions, and systemic therapies, as well as transitions across providers, services, and diagnostic procedures. During the initial phase of the pandemic, oncology care pathways were disrupted globally, with documented delays in diagnosis and treatment linked to increased mortality and higher long-term treatment costs [12, 13]. For instance, the economic impact of delays in melanoma diagnosis in Europe alone was estimated at over $7.6 billion [14]. Concurrently, emergency department visits by cancer patients declined in multiple countries, likely due to a combination of system-level restrictions and patient hesitancy [15]. Unlike many other countries, where oncology activity declined sharply, the Swiss healthcare system was comparatively effective in maintaining access to essential treatments for cancer patients. A recent cohort study using Swiss national cancer registry finds no significant effect of the COVID-19 pandemic on cancer short-term patient outcomes, including survival and the distribution of disease stage [16]. Another study showed that Swiss hospitals safeguarded cancer patients during the pandemic, ensuring a relative stability of cancer patient admissions [7]. Nevertheless, empirical evidence remains limited on how this phenomenon affected resource allocation relative to non-cancer patients and how pandemic disruptions reshaped resource distribution across hospital clinical services.

Understanding how hospital resources were reallocated during a public health emergency is critical for evaluating system resilience and preparedness. Hospital cost data provides a direct reflection of resource utilization, unlike reimbursement claims, which are often shaped by negotiated tariffs, insurer policies, or regulatory frameworks. This is particularly relevant in the decentralized Swiss health system, where hospital tariffs vary across both cantons and hospitals. In Switzerland, inpatient hospital care is reimbursed through nationally uniform DRG case groups (SwissDRG [17]) combined with hospital-specific base rates negotiated with insurers and approved by the cantonal health authorities resulting in inter-hospital tariff variation. Costs and cost-per-day are widely accepted, quantifiable indicators of care intensity, derived from internal hospital accounting systems [18, 19].

Empirical evidence on how hospital costs evolved during the pandemic from the hospital perspective remains limited. Most published data come from the United States and focus on COVID-19-specific admissions, estimating median inpatient costs ranging from $11,267 to $12,046, with substantially higher costs for ICU or ventilated patients [20–22]. Considering reimbursements, data from the Medicare program indicate that the average fee-for-service payment per COVID-19 hospitalization was $25,464 during the January-June 2020 period [23]. Available data suggest that COVID-19 led to a substantial increase in hospitalization costs across most countries [24–26]. Among U.S. cancer patients, this trend was partly driven by increased use of high-cost inpatient care for those diagnosed with COVID-19 [27]. In Switzerland, no studies have yet explored the impact of the pandemic on the costs of hospital stays, nor on the potential reallocation of resources across hospital services.

To address this gap, this study leverages nationwide hospital discharge and cost accounting data from Swiss hospitals to examine how hospital costs evolved during the COVID-19 pandemic. These data provide detailed cost breakdowns across services, enabling a nuanced analysis of resource allocation shifts during the pandemic, in response to the organizational pressures it imposed. This study provides the first disaggregated assessment of hospital cost structures and compares trends between cancer and non-cancer patients, raising both empirical and ethical considerations that are central to equitable resource allocation. Specifically, this study investigates variations in both total and component-specific hospital costs across pandemic phases including pre-lockdown, lockdown, and post lockdown periods. Additionally, it explores changes in hospital revenues relative to costs to better understand the financial impact of the pandemic on hospitals.

## Method

### Data sources

This study follows the Observational Routinely-collected health Data (RECORD) reporting guidelines [28] and is based on routinely collected inpatient data from acute somatic care hospitals in Switzerland, covering the period from January 2017 to December 2021. Data were obtained from the Swiss Federal Statistical Office (FSO), which compiles standardized administrative discharge data for monitoring public health, supporting resource planning, ensuring quality-of-care control, and regulatory oversight [29].

Two FSO datasets contain inpatient records. The first dataset, the Swiss Medical Statistics of Hospitals, is a mandatory annual survey that compiles sociodemographic and clinical data on all inpatients admitted to Swiss hospitals [29]. The second dataset, the Hospital Case Cost Statistics, complements the first by providing detailed stay-level costs derived from hospitals’ analytical accounting systems [30]. Costs are reported per stay and subdivided into accounting categories. Participation in the Hospital Case Cost Statistics is voluntary and limited to a subset of acute somatic care hospitals; the data support the annual revision of the Swiss DRG system. The Hospital Case Cost Statistics does not include patients with complementary private insurance. About 67% of stays in the Swiss Medical Statistics appear in the Hospital Case Cost Statistics each year over the five-year period analyzed. Cost estimates in this study are derived from the Hospital Case Cost Statistics dataset, with access to the anonymized data granted by the FSO under a formal data-sharing agreement.

### Medical and costs data

The dataset comprises individual stays, unlinked records that include patient characteristics (e.g., age in five-year groups and sex), clinical information (e.g., ICD-10 diagnoses [31]), length of stay (LOS), in-hospital death, and accounting-based cost data. It also includes temporal and geographic information (e.g., month of admission, hospital canton) and hospital status.

Cost data are defined by the REKOLE (Revision der Kostenrechnung und Leistungserfassung) cost accounting system, which is a nationally recognized standard for Swiss hospitals. REKOLE sets standardized guidelines ensuring that all Swiss hospitals calculate their costs using a consistent and comparable methodology. It separates costs directly related to clinical activity (direct costs) from those related to infrastructure, investments, and information technology (indirect costs also called overhead). In a first step, overhead costs are allocated to clinical service domains according to allocation keys such as surface area of premises and full-time equivalents. In a second step, clinical costs (including overhead) are attributed to patient cases based on cost drivers such as operating time, nursing scores, and tariff points. The costs of implants and medications are allocated directly to the hospital stay. Costs are calculated annually by each institution. For a given hospital, and fiscal year, cost drivers should remain fixed. These cost drivers enable the allocation of expenses across service units and the subsequent classification into cost categories, as well as the calculation of total costs associated with each hospital stay [32]. Categories correspond to distinct service domains and include, for example, nursing care, ICU, emergency services, operating room, and imaging exams [30]. A detailed description of cost categories is provided in Appendix 1.

### Population, outcomes and additional study variables

The study examined inpatient cases of adults (≥ 18 years) receiving acute somatic care. Moreover, this study focuses on patients with cancer and how their hospital management was affected by the pandemic. Accordingly, the analysis encompasses all cancer-related hospital admissions, irrespective of whether the disease was the primary reason for hospitalization or was a contributing factor to complications. Consequently, and based on the FSO admission coding manual, patients were assigned to the cancer group if a cancer diagnosis appeared in the primary or secondary diagnosis fields. This ensures that the majority of cancer patients are included. All other admissions were classified as non-cancer cases. Hospital stays were then categorized as cancer-related or non-cancer-related based on the presence or absence of ICD-10 diagnosis codes for malignant neoplasms. The full list of codes and classification rules used to define cancer-related stays are available in Appendix 2.

The primary outcome of the study is the total cost per hospital stay, measured in Swiss francs (CHF), which reflects the actual expenditures incurred by the hospital. To better understand the factors contributing to cost variability, costs are further disaggregated into six specific cost subcategories: Emergency Service (ES), Intensive Care Unit (ICU), Operating Room (OR), physician-related costs (capturing the effect of physician staffing resources), nurse-related costs (capturing the effect of nursing staffing resources), and imaging-related costs. This breakdown allows for detecting changes in resource allocation during the pandemic’s acute phase. Additional details regarding the construction of the outcome measures are provided in Appendix 1.

The secondary outcome is the revenue received by the hospital for each inpatient stay. Revenue is estimated based on the Diagnosis-Related Group (DRG) assignment for the stay and the canton-level base rate. Due to data anonymization, hospital-specific DRG base rates were not available. Instead, this study uses the minimum and maximum tariffs publicly reported for each canton to define a plausible interval for the actual revenue received per case. Details regarding the construction of the revenue outcome are provided in Appendix 1.

All monetary values were inflation-adjusted to real December 2021 CHF using the monthly Swiss consumer price index.

Covariates include age group, sex, LOS, comorbidity burden during the hospitalization (assessed using Charlson Comorbidity Index categories, excluding cancer-related comorbidities), in-hospital mortality, month of admission, hospital canton, and university hospital status. University hospitals were identified separately due to their higher ICU capacity and their central role during the COVID-19 response. Accounting for covariates is important to reduce confounding and ensure that observed associations during the lockdown (and post-lockdown) reflect accurate relationships rather than differences in patient characteristics, geographic location, or hospital context. Details on the construction of the covariates are provided in Appendix 2.

### Analytic approach

Data were grouped into three COVID-19-related time periods: pre-lockdown (January 2017 to February 2020), lockdown (March 2020 to May 2020), and post-lockdown (June 2020 to August 2021). The lockdown period was defined as all months that included any part of the national restriction phase. Switzerland implemented a federal ban on non-urgent elective surgeries, together with a national lockdown, on March 16, 2020. Restrictions were lifted in phases: elective surgeries were reauthorized and some stores reopened on April 27, followed by a broader reopening on May 11. This three-month definition, capturing the gradual reopening of schools, shops, and social activities, is broader than the strict six-week period during which hospitals were instructed to postpone all elective procedures. While some Swiss studies used the strict six-week window [8, 9], this broader definition is consistent with other national work [7].

First, a descriptive analysis was performed. Average monthly values for total and daily costs were computed and stratified by cancer status and time period. Comparisons of monthly averages were made using t-tests with 95% confidence intervals. Daily costs were calculated by dividing total costs by LOS, serving as a proxy for care intensity [19, 24, 33]. Second, monthly trends in average costs and average daily costs were plotted separately for cancer and non-cancer patients to assess differences in both timing and magnitude of cost fluctuations during the pandemic. Third, a difference-in-differences analysis was conducted to estimate the differential impact of the pandemic on hospital costs for cancer versus non-cancer patients, while controlling for potential confounders not accounted for in the descriptive approach. Although both groups were exposed to the lockdown, methodological literature supports applying DiD when treatment intensity varies across groups rather than being strictly binary [34, 35]. Using non-cancer patients as a reference isolates the specific impact of admission decisions for cancer patients during the lockdown. The regression models used individual-level data and estimated interaction effects between time period (pre-lockdown, lockdown, post-lockdown) and cancer status, under the assumption of parallel pre-pandemic trends between groups (assessed in Appendix 3) [36]. Cost outcomes were log-transformed to address skewness [37]. Robust regression using Huber weighting estimators was applied to mitigate the effect of outliers [38]. Models include month, post-lockdown indicator, comorbidity, in-hospital mortality, hospital type, LOS, sex, and fixed effects for age and canton of hospital. In addition, the regression is adjusted for a 12-month autocorrelation. For subcategory analyses, models are restricted to stays with non-zero cost values for the specific component (e.g., ICU costs analyzed only among ICU users) to avoid problems with zero inflation. Adding a constant to zero values was avoided, in line with best practices for log-transformed cost data [39]. Adjusted marginal effects were computed for all models to facilitate interpretation. These average marginal effects represent the percentage change in cost associated with cancer diagnosis, stratified by period, while holding other covariates constant [40]. More information on methodological aspects is provided in Appendix 3.

Finally, estimated revenues and costs were compared over time to evaluate potential mismatches in hospital financial performance. Revenue intervals, based on minimum, maximum, and midpoint DRG base rates (where midpoint is calculated as (maximum base rate + minimum base rate)/2), were assessed alongside cost trends for each patient group.

Statistical significance was defined as *p* < 0.05, and results exceeding this threshold were considered non-significant. All analyses were conducted using STATA software (Stata/SE 18.0 for Windows (64 bit x86-64)).

## Results

### Descriptive statistics

After data cleaning, the final study dataset comprised 3,944,239 hospital stays covering the period from January 2017 to August 2021, where cancer patients represent 10% and non-cancer patients 90% of the stays. Data cleaning procedures involved eliminating duplicate entries and excluding hospitalizations with implausible or missing values, such as negative LOS or zero total cost.

**Fig. 1:**
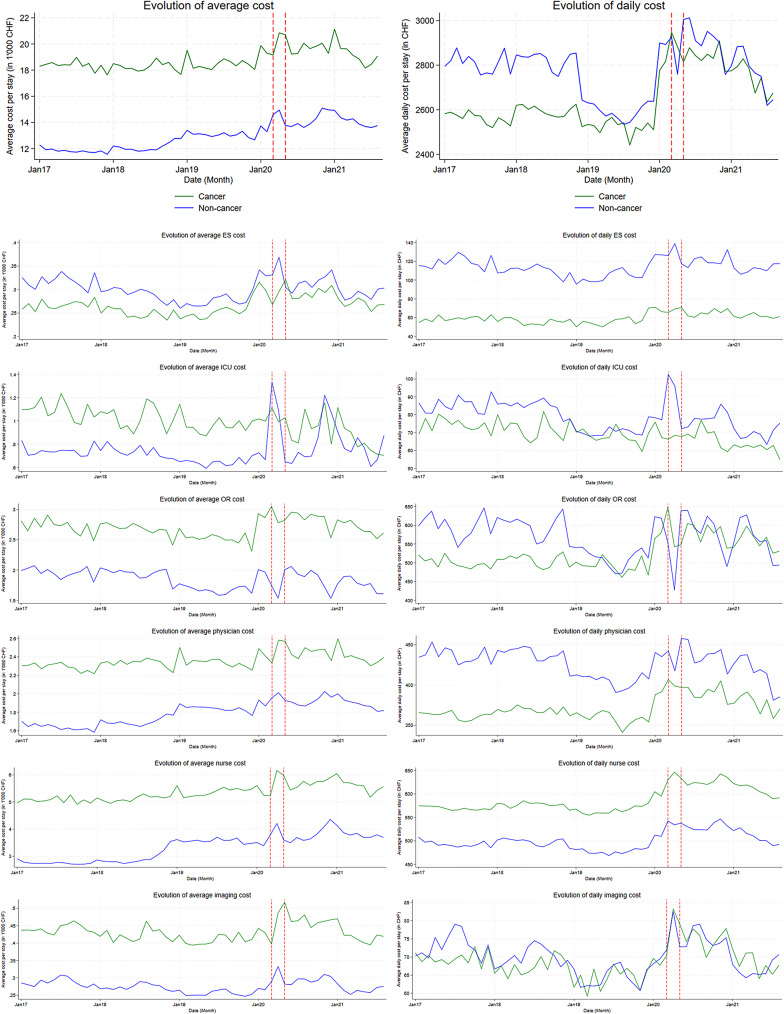
Trends in the average costs per stay (in 1,000 CHF) and daily average costs per stay, for cancer and non-cancer patients in Switzerland between January 2017 and August 2021. Red dashed vertical lines mark key time points: March 2020 (end of the pre-lockdown period), and May 2020 (beginning of the post-lockdown period)

The descriptive statistics are summarized in Table 1, while Fig. 1 illustrates the monthly cost trends. The monthly average number of admissions was 68,917 before the lockdown; during the lockdown admissions declined by 10.5% overall with a 3.9% drop among cancer patients and an 11.2% drop among non-cancer patients.

The average cost per hospital stay was CHF 13,044 during this baseline period, with cancer patients exhibiting higher average costs (CHF 18’419) than non-cancer patients (CHF 12,404). During the lockdown period, the overall average cost per stay increased by 15.7% (CHF + 2,049, 95% CI [1,353; 2,746]) compared to the pre-lockdown baseline. In the post-lockdown period, costs remained 12.7% higher than pre-lockdown levels (CHF + 1,654, 95% CI [1,316; 1,992]).

Across subcategories, the lockdown period was associated with significant increases in average ES costs (+ 12.6%, CHF + 37, 95% CI [10; 65]), ICU costs (+ 38.2%, CHF + 283, 95% CI [172; 395]), nurse services costs (+ 22.8%, CHF 759, 95% CI [331; 1,186]), and imaging costs (+ 11.1%, CHF 132, 95% CI [11; 53]).

In the post-lockdown period, average ICU costs remained elevated (+ 9.9% CHF + 73, 95% CI [14; 132]), as did nurse costs (+ 20.4%, CHF + 678, 95% CI [480; 875]). These changes reflect persistent shifts in resource-use patterns following the acute phase of the pandemic.

Subgroup analyses revealed that both groups experienced significant cost increases. For cancer patients, average stay costs increased by 9.9% (CHF + 1,823, 95% CI [1,218; 2,428]) during the lockdown and by 5.5% (CHF + 1,011, 95% CI [677; 1,345]) post-lockdown. For non-cancer patients, the corresponding increases were 16.3% (CHF + 2,027, 95% CI [1,271; 2,785]) and 14.0% (CHF + 1’735, 95% CI [1,371; 2,100]), respectively. The descriptive statistics, capturing the unadjusted effect, show a more pronounced relative cost increase during lockdown and post-lockdown among non-cancer patients compared to cancer patients. However, these results do not account for potential confounders, which are controlled for in the subsequent DID analysis.

During the lockdown, a decline in average daily costs was more pronounced among non-cancer patients, particularly in the operation room, whereas ICU costs for this group increased more markedly. A similar descriptive analysis was performed for university hospitals, in Appendix 2, revealing comparable but more pronounced trends.

**Table 1:** Monthly averages of admissions and costs for cancer and non-cancer patients across pre-lockdown, lockdown and post-lockdown periods

Monthly averages for cancer and non-cancer patients							
	Pre-lockdown (Prl) (26 months)	Lockdown (L) (3 months)			Post-lockdown (PoL) (15 months)		
	Mean	Absolute and relative mean difference		95% CI	Absolute and relative mean difference		95% CI
	**PrL**	$\:\varDelta\:\:(\mathbf{L}-\mathbf{P}\mathbf{r}\mathbf{L})$	$\:\frac{\mathbf{L}-\mathbf{P}\mathbf{r}\mathbf{L}}{\mathbf{P}\mathbf{r}\mathbf{L}}$		$\:\varDelta\:\:(\mathbf{P}\mathbf{o}\mathbf{L}-\mathbf{P}\mathbf{r}\mathbf{L})$	$\:\frac{\mathbf{P}\mathbf{o}\mathbf{L}-\mathbf{P}\mathbf{r}\mathbf{L}}{\mathbf{P}\mathbf{r}\mathbf{L}}$	
Admissions (N)	68,917	-7,156	*-10.4%*	[-15,249; 937]	7,090	*10.3%*	[3,391; 10,790]
**Costs per stay (CHF)**	**13**,**044**	**2**,**049**	***15.7%***	**[1**,**353; 2**,**746]**	**1**,**654**	***12.7%***	**[1**,**316; 1**,**992]**
Emergency costs per stay (CHF)	293	37	*12.6%*	[10; 65]	8	*2.7%*	[-5;21]
ICU costs per stay (CHF)	741	283	*38.2%*	[172; 395]	73	*9.9%*	[14;132]
Operation room costs per stay (CHF)	1,244	-45	*-3.6%*	[-164; 74]	-33	*-2.7%*	[-92; 26]
Physician costs per stay (CHF)	1,951	-58	*-3.0%*	[-241; 126]	-40	*-2.1%*	[-131; 51]
Nurse costs per stay (CHF)	3,324	759	*22.8%*	[331; 1,186]	678	*20.4%*	[480; 875]
Imaging costs per stay (CHF)	288	32	*11.1%*	[11; 53]	9	*3.1%*	[-2; 19]
**Monthly averages for cancer patients**							
Admissions (N)	7,308	-286	*-3.9%*	[-996;423]	714	*9.8%*	[362; 1,067]
**Costs per stay (CHF)**	**18**,**419**	**1**,**823**	***9.9%***	**[1**,**218; 2**,**428]**	**1**,**011**	***5.5%***	**[677; 1**,**345]**
Emergency costs per stay (CHF)	259	36	*13.9%*	[13; 58]	21	*8.1%*	[10; 32]
ICU costs per stay (CHF)	1,027	17	*1.7%*	[-96; 132]	-140	*-13.6%*	[-209; -72]
Operation room costs per stay (CHF)	1,656	229	*13.8%*	[56; 402]	111	*6.7%*	[23; 199]
Physician costs per stay (CHF)	2,328	166	*7.1%*	[85; 248]	90	*3.9%*	[51; 130]
Nurse costs per stay (CHF)	5,199	591	*11.4%*	[330; 851]	440	*8.5%*	[321; 559]
Imaging costs per stay (CHF)	425	43	*10.1%*	[14; 72]	16	*3.8%*	[2; 30]
**Monthly averages for non-cancer patients**							
Admissions (N)	61,609	-6,870	*-11.2%*	[-14,325; 586]	6’376	*10.3%*	[2,999; 9,752]
**Costs per stay (CHF)**	**12**,**404**	**2**,**027**	***16.3%***	**[1**,**271; 2**,**785]**	**1**,**735**	***14.0%***	**[1**,**371; 2**,**100]**
Emergency costs per stay (CHF)	297	38	*12.8%*	[9; 67]	6	*2.0%*	[-7; 20]
ICU costs per stay (CHF)	707	317	*44.8%*	[198; 436]	98	*13.9%*	[36; 161]
Operation room costs per stay (CHF)	1,867	-104	*-5.6%*	[-295; 86]	-57	*-3.1%*	[-150; 36]
Physician costs per stay (CHF)	1,735	230	*13.3%*	[113; 347]	171	*9.9%*	[116; 226]
Nurse costs per stay (CHF)	3,100	763	*24.6%*	[303; 1’223]	708	*22.8%*	[496; 920]
Imaging costs per stay (CHF)	271	29	*10.7%*	[9; 50]	7	*2.6%*	[-3; 18]

PrL: pre-lockdown monthly average, L: lockdown monthly average, PoL: post-lockdown monthly average, 95% CI: 95% confidence interval

The average CHF/USD exchange rate between 2017 and 2021 was 0.96 [41]

The Fig. 1 shows a relatively greater decline in the average daily cost of stay for non-cancer patients during the lockdown. This trend is further supported by a more pronounced decrease in average operating room cost and a relative decline in average daily physician services costs for non-cancer patients. The figure also indicates a relative increase in average daily ICU costs for non-cancer patients during the same period.

### Results of the difference-in-differences analysis

The estimated coefficients of the DiD models are presented in Appendix 3. Results of the average marginal effects (AME) of being a cancer patient for the three study periods are presented in Table 2. The AME confirm a significantly higher baseline cost for cancer patients compared to non-cancer patients in the pre-lockdown period (+ 10.1%, 95% CI [9.8; 10.3]). Consistently, they also show significantly higher costs for cancer patients in several categories, including OR (+ 12.9%, 95% CI [12.4; 13.3]), physician services (+ 9.1%, 95% CI [8.8; 9.3]), nursing services (+ 12.6%, 95% CI [12.4; 12.8]), and imaging (+ 12.4%, 95% CI [11.7; 12.8]), but lower ICU costs (-8.0%, 95% CI [-9.5; -6.6]).

The DID interaction coefficient indicates an additional cost increase for cancer patients relative to non-cancer patients, beyond the general lockdown effect observed for both groups. The cost differential between cancer and non-cancer patients during the lockdown period is 14.9% (95% CI [14.0; 15.7]), based on AME derived from the DID estimates. This contrasts with the descriptive analysis, which suggested a smaller difference (9.9% for cancer vs. 16.3% for non-cancer patients). ICU costs for cancer patients decreased relative to non-cancer patients during the lockdown (-31.5%, 95% CI [-36.9; -26.0]), while OR costs (+ 17.6%, 95% CI [16.0; 19.3]), physician services (+ 11.2%, 95% CI [10.2; 12.2]), and nurse services (+ 15.2%, 95% CI [14.4; 16.0]) increased.

In the post-lockdown period, the cost differential between cancer and non-cancer patients remained marked (+ 13.8%, 95% CI [13.5; 14.2]). Cancer patients continued to exhibit higher physician (+ 11.7%, 95% CI [11.3; 12.1]) and nurse (+ 14.6%, 95% CI [14.3; 15.0]) costs compared to non-cancer patients, along with persistently lower ICU costs (-22.5%, 95% CI [-24.9; -20.1]).

**Table 2:** Average marginal effects of having cancer diagnosis regarding the cost outcomes

Outcome	Marginal Effect (95% CI)		
	Pre-lockdown	Lockdown	Post-lockdown
Total costs	10.1% [9.8, 10.3]	14.9% [14.0, 15.7]	13.8% [13.5, 14.2]
ES costs	-0.2% [-0.6, 0.2]	-0.4% [-1.8, 1.1]	-0.4% [-1.0, 0.2]
ICU costs	-8.0% [-9.5, -6.6]	-31.5% [-36.9, -26.0]	-22.5% [-24.9, -20.1]
OR costs	12.9% [12.4, 13.3]	17.6% [16.0, 19.3]	13.6% [12.9, 14.3]
Physician costs	9.1% [8.8, 9.3]	11.2% [10.2, 12.2]	11.7% [11.3, 12.1]
Nursing costs	12.6% [12.4, 12.8]	15.2% [14.4, 16.0]	14.6% [14.3, 15.0]
Imaging costs	12.4% [11.7, 12.8]	12.9% [11.0, 14.9]	13.3% [12.5, 14.1]

CI 95%: 95% confidence interval; ES: Emergency service; ICU: Intensive Care Unit; OR: Operation Room

### Cost versus revenue comparison

**Fig. 2:**
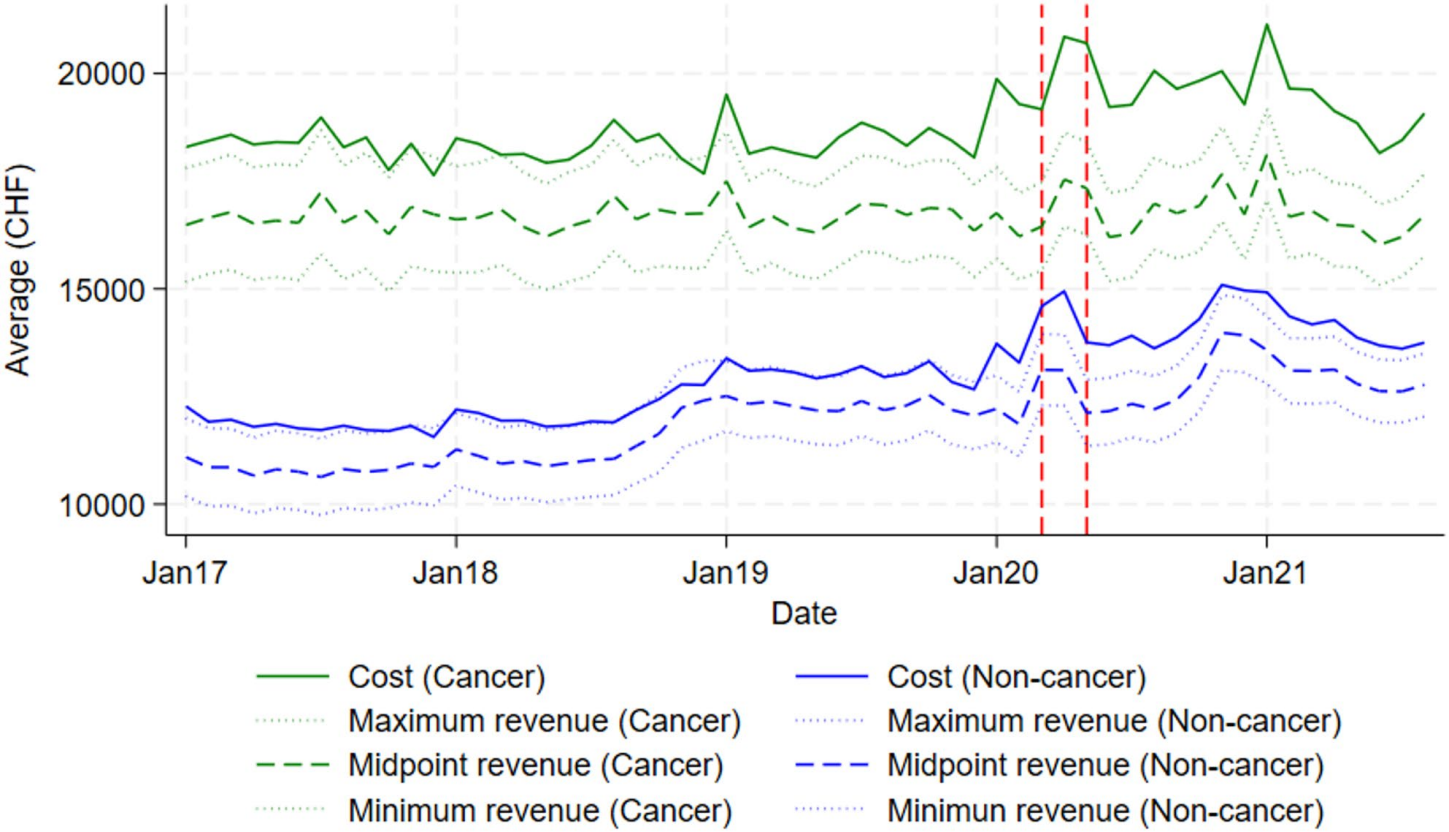
Trends in the average cost and revenue of stay for cancer and non-cancer patients between January 2017 and August 2021. Red dashed vertical lines mark key time points: March 2020 (end of the pre-lockdown period) and May 2020 (beginning of the post-lockdown period)

As shown in Fig. 2, trends in the relationship between hospital costs and revenue, indicate that DRG-based reimbursement increased for both cancer and non-cancer patients during the lockdown. However, the concurrent increase in hospital costs outpaced revenue growth, resulting in a widening gap between average costs and revenues. This divergence was especially evident among non-cancer patients, reflecting both increased complexity and changes in the case mix of admissions during the lockdown compared to the pre-lockdown period.

## Discussion

### Summary of findings

This study examined the differential impact of the COVID-19 pandemic on Swiss hospital costs for cancer versus non-cancer patients across pre-lockdown, lockdown, and post-lockdown periods. Both patient groups experienced substantial cost increases during lockdown, with overall hospital costs per stay rising by 16%, nurse costs by 23%, and ICU costs by 38%. After controlling for patient characteristics and confounding factors through DiD analysis, cancer patients showed greater cost increases than non-cancer patients, particularly in OR, physician, and nurse services, translating to an average marginal effect of 14.9% on total costs. Notably, cost patterns diverged for ICU care: while cancer patients had higher overall costs, their ICU costs increased significantly less than those of non-cancer patients (21% lower increase).

This study provides the first detailed breakdown of Swiss hospital costs by service type during COVID-19, with a focus on cancer patients. Using hospital case cost data, the analysis highlights how pandemic policies affected resource allocation, care intensity, and operational constraints, offering insights into the cost dynamics of hospital stays.

### Pre-pandemic patterns of costs

Prior to the pandemic, cancer patients incurred significantly higher costs of stay than non-cancer patients, indicating that cancer patient management is generally more resource-intensive and costly than non-cancer patient management, with the exception of critical care, where emergency services and ICU costs do not show differences between groups. Between late 2018 and early 2019, the cost of hospital stays for non-cancer patients rose, likely reflecting a higher concentration of more complex cases in inpatient settings as less severe cases shifted to outpatient care. At the same time, daily costs for non-cancer patients declined, reflecting longer hospital stays. This trend aligns with the Swiss inpatient care reform, initiated in 2018 and made mandatory in 2019, which aimed to reduce hospitalizations [42, 43].

### Lockdown period impact and resource reallocation

During the lockdown, hospital admissions decreased by 11% (Table 1) compared to the pre-lockdown period. This finding aligns with international evidence on pandemic impacts on inpatient care use [1–4]. In the Swiss context, other studies reported larger declines in the number of hospital admissions during the lockdown [7–9]. The difference between the magnitude of declines observed in this study and those reported elsewhere reflects variation in scope (procedure volumes vs. overall admissions), time frames (strict six-week lockdown vs. extended periods), methodologies (comparative baseline periods), and data sources (insurance claims vs. hospital registries). Moreover, the Swiss hospital registries may not uniformly include the same facilities, leading to discrepancies in admission counts. For instance, using the same analytical approach but different datasets, Brunner et al. reported an overall decline of 9% and 18% for cancer and non-cancer patients respectively whereas this study observed decreases of approximately 4% and 11%, respectively. Importantly, the relative proportion of cancer to non-cancer patients in both Swiss hospital registries (Swiss Medical Statistics of Hospitals [7] and Hospital Case Cost Statistics) remains similar across datasets, supporting the comparability of the findings.

The average cost of hospital stays in Switzerland was 15.7% higher during the lockdown than in the pre-lockdown period (CHF + 2,049, 95% CI [1,353; 2,746]). This increase remained statistically significant after adjusting for covariates in the DiD model. Several factors likely contributed to this rise: the surge in COVID-19 patients requiring critical care, increased personnel costs (extended hours, additional staffing), and higher protective equipment expenditures. The observed increase in ICU costs during the lockdown likely reflected the substantial burden of severe COVID-19 cases and the inherently resource-intensive nature of critical care, such as mechanical ventilation, continuous monitoring, and complex pharmacological management [20, 44].Meanwhile, average OR costs declined, reflecting fewer surgeries performed during the lockdown period due to the federal ban on non-urgent elective surgical procedures [45]. This effect was more pronounced in university hospitals, which served as primary COVID-19 centers (Appendix 2). This sharper decline likely reflected both greater reallocation toward pandemic response and the deferral of surgeries that are typically high in complexity and cost, which are characteristic of academic medical centers. These findings are also consistent with the sharp reduction in elective surgeries observed in Switzerland during the spring pandemic wave, with total knee and hip replacements dropping by 85% and 76%, respectively. In contrast, time-sensitive emergency admissions decreased far less, with stroke and myocardial infarction admissions falling by only 14% and 9% [9].

### Unadjusted versus adjusted results

After adjusting for patient and hospital characteristics, the lockdown was associated with a larger increase in costs for cancer patients compared to non-cancer patients. This finding contrasts with the unadjusted descriptive analysis showing larger increases for non-cancer patients (16.3% vs. 10%). This reversal stems from substantial confounding effects. By adjusting for LOS, comorbidities, and in-hospital mortality, the DID model isolates the effect of cancer status from underlying disease severity, revealing that among patients with broadly comparable characteristics, cancer patients were relatively more resource-intensive during the lockdown.

### Differential resource allocation between cancer and non-cancer patients

Higher adjusted per-stay costs, reflecting greater use of OR, physician, and nursing services, suggest that cancer patients were assigned relatively higher attention during the study period. This likely reflects the time-sensitive nature of cancer treatments, for which delays can compromise outcomes and disrupt ongoing therapeutic pathways, combined with the complex interaction between COVID-19 and cancer comorbidities. These findings align with administrative directives that explicitly emphasized prioritizing cases based on clinical urgency, such as those involving cancer, during the pandemic. Other studies based on Swiss data also found that cancer patients experienced a much smaller decline in hospital admissions than other chronic-disease patients, indicating that care for vulnerable patients was explicitly maintained as a priority during this period in Switzerland [7, 46].

The increased difference in ICU costs between cancer and non-cancer patients during lockdown (21% lower increase for cancer patients) may be attributed to sustained and intensified provision of treatments to cancer patients in ward settings, as a preventive measure to limit transfers to ICU. This shift likely resulted in lower ICU care intensity for cancer patients compared to pre-lockdown levels. The divergent patterns between total hospital costs and ICU costs show that resources were strategically allocated to maintain cancer ward-level care, while ICU resources were predominantly directed toward managing critically ill patients, likely including many with severe COVID-19 complications. The 44.8% increase in ICU costs per stay for non-cancer patients (compared to 1.7% for cancer patients) in descriptive statistics reflects the extraordinary resource demands of COVID-19 critical care, an increase that is relatively greater among patients who had a lower likelihood of ICU admission prior to the pandemic. Moreover, prevalence of COVID was probably higher for non-cancer patients, as individuals with chronic conditions were strongly advised to protect themselves and generally adhered better to confinement measures.

### Persistent post-lockdown effects

The cost of stay remained higher than pre-lockdown levels nine months after the lockdown, indicating that the pandemic increased the intensity of care across all the subcategories investigated. This finding confirmed that hospital costs in Switzerland followed a similar trend to those observed globally [47–51], with ICU costs remaining higher in the post-lockdown phase [25, 49]. In Switzerland, this sustained increase was likely not only due to the continued demand for critical care but also reflected the intensified involvement of healthcare staff, as evidenced by the rise in nursing costs. During the post-lockdown period, cancer patients continued to receive more resources and time, as evidenced by higher costs of hospital stays and physician services. Continued attention to cancer patients was reflected in the observed pattern of decreased ICU costs, suggesting a shift in hospital practice toward more proactive and preventive care for this group. Several mechanisms likely contributed to the sustained elevation in costs: prolonged implementation of infection control measures that continued beyond the acute lockdown period; extended procedure duration under pandemic protocols; and backlogs of deferred care requiring more intensive treatment due to disease progression during delays [52]. For cancer patients, the persistent cost differential may be attributable to delayed screening and diagnostics, which led to more advanced disease at presentation, as well as treatment modifications implemented during lockdown that required subsequent protocol adjustments.

### Financial implications and policy response

The crisis imposed substantial financial strain, disrupting both operational and revenue models. Although demand for ICU services surged, this increase did not result in a proportionate rise in hospital revenues in Switzerland under the standard DRG payment model, given the exceptional volume and intensity of resources used. ICU care is inherently costly, requiring specialized staff, advanced equipment, and extended patient stays, all of which substantially increase operational expenses. At the same time, hospitals experienced a marked decline in revenue due to the postponement or cancellation of elective and non-urgent procedures, which are typically more profitable and help offset the costs of complex care.

This situation prompted Swiss policymakers to allocate exceptional funding. In 2020, Swiss cantonal authorities injected CHF 1.106 billion into hospitals to help absorb the financial shock related to the decrease in admissions during the COVID-19 pandemic, the management of COVID cases and to maintain service quality [53]. This funding was essential to support the surge in resource demand during the pandemic. However, as shown in the U.S., such financial support alone may not be sufficient to incentivize more resource-intensive care, highlighting the need for better-targeted strategies. U.S. hospitals, which are predominantly private, often responded to revenue shortfalls by cutting costs through layoffs, furloughs, and salary reductions [54]. In Switzerland, the widening cost-revenue gap was especially pronounced for non-cancer patients, potentially reducing incentives to admit or prioritize these less profitable cases.

### Policy implications

This study provides evidence that pandemic-specific reimbursement mechanisms are essential to account for elevated costs during health crises. Swiss hospitals operating under prospective DRG-based payment face significant financial pressure when per-case costs rise. Without payment system adjustments such as temporary pandemic-related modifiers or retrospective cost reconciliation, payment structures do not provide the financial resources to maintain resource-intensive services precisely when they are most needed.

This study allows to check whether Swiss hospitals allocated resources to patients with the greatest need. This indicates that COVID-19 and cancer were major cost drivers, suggesting that these areas received particular attention to ensure their needs were met. This aligns with Switzerland’s epidemiological context, where hospitals were not overwhelmed and could avoid prioritizing among critically ill patients, thereby maintaining care for those most in need. Existing Swiss literature corroborates this, noting that patient prioritization during lockdown was primarily based on medical emergency [46]. However, without additional clinical outcome data, it remains unclear whether the observed allocation patterns optimized population health or inadvertently created barriers to necessary non-cancer care. Future policies should incorporate equity monitoring, especially during health crises, by tracking access metrics stratified by patient characteristics. Such measures would support efficient allocation and correct inequities that may arise, an internationally recognized challenge during the COVID-19 [55].

In response, ICU capacity was expanded to accommodate patients with severe illness, while the findings of this study suggest that maintaining sustained ward-level capacity for conditions such as cancer remained feasible in Switzerland. However, pandemic preparedness should include explicit protocols identifying services that cannot be safely deferred and whose patients are unlikely to require intensive care.

### Limitations

First, COVID-19 diagnosis could not be ascertained due to uncertainties regarding the accuracy and completeness of COVID-19 diagnostic data during the early phase of the pandemic. COVID-19 cases were often reported retrospectively, without systematic testing, and with no ICD-10 code for COVID-19 available in the early months of the pandemic. Thus, the study did not distinguish between COVID-19 and non-COVID-19 cases. COVID-19 diagnosis remains a significant confounder, that participates to explain the extraordinary costs of ICU for non-cancer patients in crude comparisons. Although in terms of statistical variables, it was not possible to separate COVID disease and lockdown policy effects, the DID approach mitigates this limitation as it first adjusts for LOS and takes into account the effects of the lockdown policies, that coincided with the first wave of the COVID-19 prevalence.

Second, the study did not use a standardized comorbidity index to account for patient health complexity, due to the lack of comprehensive linkage between hospital stay data across the study years. Instead, the index used to account for comorbidity relied on the presence of comorbidities reported during the actual hospital stay without considering previous stays. This approach may underestimate the burden of comorbid conditions, particularly if some relevant comorbidities were not documented.

Third, as participation in the Hospital Case Cost Statistics dataset is not systematic for all hospitals, this introduces potential selection bias. Smaller hospitals, often operating with less efficient accounting systems, tended to provide incomplete or no cost data. As a result, our analysis is largely restricted to larger hospitals, which were more likely to play a regional role during the pandemic. This may explain why the observed decrease in admissions during the lockdown was smaller in this analysis than in the literature. Nevertheless, the proportion of cancer and non-cancer patients remains partly consistent with studies using the full dataset, (11.3% of cancer patients in the present study vs. 11.7% [7]), and the cost information derived from this sample can be prudently generalized to all Swiss hospitals.

## Conclusion

The COVID-19 pandemic fundamentally altered Swiss hospital cost structures, with heterogeneous effects across hospital services. This study highlights significant increases in care intensity, particularly for cancer patients. Simultaneously, the concentration of ICU cost increases among non-cancer patients, likely driven by COVID-19 critical care demands, illustrates how the pandemic exerted uneven pressure on different components of healthcare delivery. The observed substantial and persistent cost increases, combined with evidence that cancer care maintained relative resource intensity, have important implications for healthcare financing and future pandemic preparedness to ensure equitable access to essential services during public health emergencies. Explicit protocols for maintaining time-sensitive services like cancer management, coupled with payment mechanisms that accommodate elevated costs, are essential to optimize both immediate pandemic response and to preserve essential care continuity.

## Supplementary Information

Below is the link to the electronic supplementary material.

Supplementary Material 1

Supplementary Material 2

Supplementary Material 3

## Data Availability

The dataset analyzed for the current study is not publicly available due to ethical and research governance requirements for data access.

## Abbreviations

AME: Average Marginal Effect
CI: Confidence Interval
DRG: Diagnosis-Related Group
ES: Emergency Service
FSO: Federal Statistical Office
ICD-10: International Classification of Diseases
ICU: Intensive Care Unit
LOS: Length Of Stay
OR: Operating Room
