# Supplementary material for: Costs of hospital stays in Switzerland during the COVID-19 pandemic: a comparative analysis between cancer and non-cancer patients
Source: BMC Health Serv Res. 2026 Apr 24;26:934. doi: 10.1186/s12913-026-14585-0 (PMC13343702; doi:10.1186/s12913-026-14585-0)
Supplement: Supplementary file 1 — Supplementary Material 1 [file 12913_2026_14585_MOESM1_ESM.docx]

**Appendix 1, hospital costs and revenue:**

***Hospital cost***

The cost variable in the dataset represents the full cost per case for inpatient stays in Swiss acute somatic hospitals. SwissDRG is responsible for collecting these data and transmitting them to the Swiss Federal Statistical Office in accordance with the Law. Hospital participation to the data collection is voluntary.

Costs per hospital stay are disaggregated into detailed accounting categories, primarily including personnel costs and operating expenses. Case-level costs are computed using a standardized methodology that integrates patient-level data, service utilization, and institutional cost information.

Cost of a stay

- The cost of stay represents the total cost incurred by the hospital for a patient during the stay. It is the most aggregated cost variable, encompassing all expenses generated during the stay. This include both: controllable costs (medical procedure, medication, …) and non-controllable costs (administrative overhead, building maintenance, amortization, …) [25].

The following cost categories represent sub-aggregations derived from this integrated data.

ES cost for a stay

- Breakdown of cost specifically incurred in the emergency service (ES), including both ES utilization and ES physician cost for both controllable and non-controllable costs.

ICU cost for a stay

- Breakdown of cost specifically incurred in the intensive care unit (ICU), including both ICU utilization and ICU physician cost for both controllable and non-controllable costs.

OR cost for a stay

- Breakdown of cost specifically incurred in the operation room (OR), including both OR utilization (including anesthesia costs) and OR physician cost for both controllable and non-controllable costs.

Physician cost for a stay

- Breakdown of physician-related costs not captured within other cost categories, including both controllable and non-controllable costs.

Nurse cost for a stay

- Breakdown of nurse-related costs, including both controllable and non-controllable costs.

Imaging cost for a stay

- Breakdown of costs associated with imaging services, including both controllable and non-controllable costs.

***Hospital revenue***

Hospital revenue is defined as the Diagnosis Related Group (DRG) payment assigned at the end of each hospital stay. As this information was not directly available in the dataset, it was computed using the Swiss DRG grouper [51], which calculates a stay’s value based on diagnostic codes and length of stay. This estimated value has then to be adjusted by multiplying it with hospital-specific base rates to approximate actual revenue. Due to data anonymization requirement, these hospital-specific base rates were not available. To address this limitation, a revenue range was constructed using the minimum and maximum base rates from each canton This method provides a reliable interval within which the actual revenue likely falls, accounting for potential variation introduced by anonymization.

Revenue was estimated for each individual hospital stay. Since the exact revenue depends on hospital-specific base rates, three variables were derived to capture the possible range of values:

- - Maximum revenue: The upper bound of the estimated revenue range
  - Average revenue: The midpoint of the estimated revenue range
  - Minimum revenue: The lower bound of the estimated revenue range
