# Supplementary material for: Costs of hospital stays in Switzerland during the COVID-19 pandemic: a comparative analysis between cancer and non-cancer patients
Source: BMC Health Serv Res. 2026 Apr 24;26:934. doi: 10.1186/s12913-026-14585-0 (PMC13343702; doi:10.1186/s12913-026-14585-0)
Supplement: Supplementary file 2 — Supplementary Material 2 [file 12913_2026_14585_MOESM2_ESM.docx]

**Appendix 2, variable list**

Time Periods:

- Pre-lockdown: January 2017 to February 2020 (38 months)
- Lockdown: March 2020 to May 2020 (3 months)
- Post-lockdown: June 2020 to August 2021 (15 months)

Patient Categories:

- Adult cancer patients: Hospital stays involving adult patients (age ≥ 18) with at least one cancer diagnosis according to ICD-10 (see “code used to identify variables”)).
- Adult non-cancer patients: Hospital stays involving adult patients (age ≥ 18) without any cancer diagnosis according to ICD-10 (see “code used to identify variables”).

Covariates:

- Age of Patient: Categorical variable indicating the age group, divided into 5-year age bands
- Gender: Whether the patient is male (binary variable: 1 = male, 0 = not male).
- Length of Stay (LOS): Number of days a patient spends in the hospital during a single stay (expressed as a continuous variable in days).
- Comorbidities: Presence of one or more additional medical conditions co-occurring with the cancer condition. The comorbidities considered are acute myocardial infraction, congestive heart failure, peripheral vascular disease, cerebrovascular disease, dementia, Chronic obstructive pulmonary disease, rheumatoid disease, peptic ulcer, diabetes, diabetes with complications, hemiplegia, paraplegia, renal disease, mild liver disease, moderate liver disease, severe liver disease, and AIDS. These diseases are identified using ICD-10 codes (see “code used to identify variables”) (binary variable: 1 = one or more comorbidities, 0 = no comorbidities).
- In-hospital death: Whether the patient died during the hospital stay (binary variable: 1 = death, 0 = no death).
- Hospital canton: Categorical variable representing the region (canton) of the hospital where the admission occurred
- University Hospital: Whether the stay took place in a university hospital (binary variable: 1 = university hospital, 0 = non-university hospital).
- 12-Month indicator: represents the month of admission, coded from 1 to 12. This variable is included to account for potential seasonal effects on admission patterns.

Summary of Variable Types:

- Binary Variables: In-hospital death, Comorbidities, Gender, University Hospital.
- Categorical Variables: Age of Patient, Canton of the hospital, 12-Month indicator.
- Continuous Variables: Length of Stay (LOS).

***Codes used to identify variables***

ICD-10 codes for malignant neoplasms: "C00" -"C97"

Comorbidities:

- Acute myocardial infarction (AMI)I: "I21", "I22", and "I252"
- Congestive heart failure (HF): "I099", "I110", "I130", "I132", "I255", "I420", "I425", "I426", "I427", "I428", "I429", "I43", "I50", and "P290"
- Peripheral vascular disease: "I70", "I71", "I731", "I738", "I739", "I771", "I790", "I792", "K551", "K558", "K559", "Z958", and "Z959"
- Cerebrovascular disease: "G45", "G46", "I60", "I61", "I62", "I63", "I64", "I65", "I66", "I67", "I68", "I69", and "H340"
- Dementia: "F00", "F01", "F02", "F03", "G30", "F051", and "G311"
- Chronic obstructive pulmonary disease (COPD): "J40", "J41", "J42", "J43", "J44", "J45", "J46", "J47", "J60", "J61", "J62", "J63", "J64", "J65", "J66", "J67", "J684", "J701", "J703", "I278", and "I279"
- Connective tissue disease: "M05", "M06", "M32", "M33", "M34", "M315", "M351", "M353", and "M360"
- Peptic ulcer disease: "K25", "K26", "K27", and "K28"
- Mild liver disease: "B18", "K73", "K74", "K700", "K701", "K702", "K703", "K709", "K713", "K714", "K715", "K717", "K760", "K762", "K763", "K764", "K768", "K769", and "Z944"
- Diabetes mellitus uncomplicated: "E100", "E101", "E106", "E108", "E109", "E110", "E111", "E116", "E118", "E119", "E120", "E121", "E126", "E128", "E129", "E130", "E131", "E136", "E138", "E139", "E140", "E141", "E146", "E148", and "E149"
- Diabetes with end-organ damage: "E102", "E103", "E104", "E105", "E107", "E112", "E113", "E114", "E115", "E117", "E122", "E123", "E124", "E125", "E127", "E132", "E133", "E134", "E135", "E137", "E142", "E143", "E144", "E145", and "E147"
- Hemiplegia or paraplegia: "G81", "G82", "G041", "G114", "G801", "G802", "G830", "G831", "G832", "G833", "G834", and "G839"
- Moderate/severe chronic kidney disease (CKD): "N18", "N19", "I120", "I131", "N032", "N033", "N034", "N035", "N036", "N037", "N052", "N053", "N054", "N055", "N056", "N057", "N250", "Z490", "Z491", "Z492", "Z940", and "Z992"
- Moderate/severe liver disease: "I850", "I859", "I864", "I982", "K704", "K711", "K721", "K727", "K729", "K765", "K766", and "K767"
- AIDS: "B20", "B21", "B22", and "B24"

***Descriptive statistics of sample***

| **Cancer and non-cancer patients** | | | | | | | |
| --- | --- | --- | --- | --- | --- | --- | --- |
|  | **Pre-lockdown**  **(26 months)** | **Lockdown**  **(3 months)** | | | **Post-lockdown**  **(15 months)** | | |
| **Monthly average** | **Mean** | **Difference** | | **CI 95%** | **Difference** | | **CI 95%** |
|  | **PrL** | $\boldsymbol{\Delta(L-PrL)}$ | $\frac{\mathbf{L-PrL}}{\mathbf{PrL}}$ |  | $\boldsymbol{\Delta(PoL-PrL)}$ | $\frac{\mathbf{PoL-PrL}}{\mathbf{PrL}}$ |  |
| Comorbidity >= 1 (N) | 22’198 | -1’648 | *-7.4%* | [-4’924; 1’629] | 3’593 | *16.2%* | [2’068; 5’119] |
| Male (N) | 31’968 | -2’852 | *-8.9%* | [-6’777;1’073] | 3’722 | *11.6%* | [1’959; 5’486]] |
| Age >= 65 and < 80 (N) | 33’708 | -4’215 | *-12.5%* | [-8’759; 330] | 3’738 | *11.1%* | [1’656; 5’821] |
| Age >= 80 (N) | 16’751 | -2’150 | *-12.8%* | [-4’562; 261] | 1’924 | *11.5%* | [802; 3’046] |
| Intensive care during the stay (N) | 4’606 | -564 | *-12.2%* | [-839; -290] | -282 | *-6.1%* | [-433; -131] |
| Admissions within a university hospital (N) | 13’090 | -2’053 | *-15.7%* | [-3’196; -910] | 327 | *2.5%* | [-228; 882] |
| Admission in the emergency service (N) | 35’093 | -1’311 | *-3.7%* | [-4’293; 1’672] | 2’724 | *7.8%* | [1’331; 4’116] |
| LOS (days) | 6.79 | 0.97 | *14.3%* | [-0.36; 2.29]] | 1.37 | *20.2%* | [0.76; 1.97] |
| In-hospital Death (N) | 1’480 | 169 | *11.4%* | [-43; 408] | 264 | *17.8%* | [119; 408] |
| **Cancer patients** | | | | | | | |
| Comorbidity >= 1 (N) | 3’051 | -35 | *-1.1%* | [-423; 353] | 500 | *16.4%* | [312; 688] |
| Male (N) | 4’108 | -119 | *-2.9%* | [-509; 271] | 398 | *9.7%* | [207; 590]] |
| Age >= 65 and < 80 (N) | 5’128 | -280 | *-5.5%* | [-837; 278] | 638 | *12.4%* | [365; 911] |
| Age >= 80 (N) | 2’152 | -119 | *-5.5%* | [381; 144] | 346 | *16.1%* | [217; 475] |
| Intensive care during the stay (N) | 645 | -84 | *-13.0%* | [-140; -29] | -53 | *8.2%* | [-86; -20] |
| Admissions within a university hospital (N) | 1’970 | -200 | *-10.2%* | [-381; -19] | 77 | *3.9%* | [-13; 167] |
| Admission in the emergency service (N) | 2’978 | -92 | *-3.1%* | [-333; 150] | 186 | *6.2%* | [68; 303] |
| LOS (days) | 8.90 | -0.16 | *1.8%* | [-0.63; 0.30] | 0.005 | *0.06%* | [-0.21; 0.22] |
| In-hospital Death (N) | 551 | 18 | *3.3%* | [-47; 85] | 50 | *9.1%* | [19; 81] |
| **Non-cancer patients** | | | | | | | |
| Comorbidity >= 1 (N) | 19’148 | -1’612 | *-8.4%* | [-4’534; 1’309] | 3’003 | *15.7%* | [1’738; 4’449] |
| Male (N) | 27’860 | -2’733 | *-9.8%* | [-6’308; 842] | 3’324 | *11.9%* | [1’731; 4’917] |
| Age >= 65 and < 80 (N) | 28’580 | -3’935 | *-13.8%* | [-7’979; 110] | 3’100 | *10.8%* | [1’262; 4’939] |
| Age >= 80 (N) | 14’598 | -2’031 | *13.9%* | [-4’207; 145] | 1’578 | *10.8%* | [570; 2’586] |
| Intensive care during the stay (N) | 3’961 | -480 | *-12.1%* | [-715; -244] | -229 | *-5.8%* | [-354; -104] |
| Admissions within a university hospital (N) | 11’121 | -1’853 | *-16.7%* | [-2’038; -868] | 250 | *2.2%* | [-225; 725] |
| Admission in the emergency service (N) | 32’115 | -1’219 | *-3.8%* | [-3’994; 1’556] | 2’538 | *7.9%* | [1’247; 3’829] |
| LOS (days) | 6.54 | 1.10 | *16.8%* | [-0.34; 2.53] | 1.53 | *23.4%* | [0.87; 2.19] |
| In-hospital Death (N) | 929 | 150 | *16.1%* | [-21; 321] | 214 | *23.0%* | [88; 339] |

PrL: pre-lockdown monthly average, L: lockdown monthly average, PoL: post-lockdown monthly average, CI: confidence interval

***Descriptive statistic of stays in university hospitals***

| **Cancer and non-cancer patients** | | | | | | | |
| --- | --- | --- | --- | --- | --- | --- | --- |
|  | **Pre-lockdown**  **(26 months)** | **Lockdown**  **(3 months)** | | | **Post-lockdown**  **(15 months)** | | |
| **Monthly average** | **Mean** | **Difference** | | **CI 95%** | **Difference** | | **CI 95%** |
|  | **PrL** | $\boldsymbol{\Delta(L-PrL)}$ | $\frac{\mathbf{L-PrL}}{\mathbf{PrL}}$ |  | $\boldsymbol{\Delta(PoL-PrL)}$ | $\frac{\mathbf{PoL-PrL}}{\mathbf{PrL}}$ |  |
| Admissions (N) | 13’090 | -2’053 | *-15.7%* | [-3’196; -910] | 327 | *2.5%* | [-228; 882] |
| **Cost for a stay (CHF)** | **18’220** | **3’616** | ***19.8%*** | **[2’842; 4’389]** | **2’268** | ***12.4%*** | **[2’093; 3’043]** |
| Emergency cost for a stay (CHF) | 473 | 53 | *11.2%* | [1; 105] | 30 | *6.3%* | [3; 59] |
| ICU cost for a stay (CHF) | 1’430 | 716 | *50.1%* | [509; 922] | 213 | *14.9%* | [93; 334] |
| Operation room cost for a stay (CHF) | 2’740 | 59 | *2.2%* | [-120; 239] | 90 | *3.3%* | [-18; 199] |
| Physician services cost for a stay (CHF) | 1’944 | 427 | *22.0%* | [255; 601] | 330 | *17.0%* | [246; 414] |
| Nurse services cost for a stay (CHF) | 4’513 | 1’046 | *23.2%* | [532; 1’560] | 992 | *22.0%* | [729; 1’256] |
| Imaging cost for a stay (CHF) | 432 | 90 | *21.3%* | [68; 113] | 50 | *11.8%* | [33; 67] |
| **Cancer patients** | | | | | | | |
| Admissions (N) | 1’970 | -200 | *-10.2%* | [-381; -19] | 76 | *3.9%* | [-13; 167] |
| **Cost for a stay (CHF)** | **24’686** | **3’147** | ***12.7%*** | **[1’823; 4’466]** | **2’568** | ***10.4%*** | **[1’871; 3’264]** |
| Emergency cost for a stay (CHF) | 326 | 43 | *13.2%* | [7; 80] | 39 | *12.0%* | [21; 57] |
| ICU cost for a stay (CHF) | 1’305 | 241 | *18.5%* | [-15; 496] | -153 | *-11.7%* | [-312; 6] |
| Operation room cost for a stay (CHF) | 2’305 | 224 | *9.7%* | [76; 373] | 156 | *6.8%* | [77; 235] |
| Physician services cost for a stay (CHF) | 3’497 | 366 | *10.5%* | [136; 596] | 284 | *8.1%* | [164; 405] |
| Nurse services cost for a stay (CHF) | 6’978 | 828 | *11.9%* | [238; 1417] | 1’052 | *15.1%* | [784; 1’321] |
| Imaging cost for a stay (CHF) | 534 | 77 | *14.4%* | [33; 122] | 55 | *10.3%* | [32; 77] |
| **Non-cancer patients** | | | | | | | |
| Admissions (N) | 11’121 | -1’853 | *-16.6%* | [-2’837; -868] | 250 | *2.2%* | [-225; 725] |
| **Cost for a stay (CHF)** | **17’076** | **3’611** | ***21.1%*** | **[2’827; 4’396]** | **2’548** | ***14.9%*** | **[2’073; 3’023]** |
| Emergency cost for a stay (CHF) | 449 | 58 | *12.9%* | [1; 115] | 30 | *6.7%* | [0; 60] |
| ICU cost for a stay (CHF) | 1’453 | 813 | *56.0%* | [576; 1’051] | 279 | *19.2%* | [153; 405] |
| Operation room cost for a stay (CHF) | 2’537 | -14 | *-0.6%* | [-207; 179] | 53 | *2.1%* | [--62; 167] |
| Physician services cost for a stay (CHF) | 1’836 | 442 | *24.1%* | [265; 620] | 325 | *17.7%* | [238; 412] |
| Nurse services cost for a stay (CHF) | 4’077 | 1’051 | *25.8%* | [513; 1’589] | 974 | *23.9%* | [699; 1’249] |
| Imaging cost for a stay (CHF) | 414 | 92 | *22.2%* | [70; 114] | 49 | *11.8%* | [32; 66] |

PrL: pre-lockdown monthly average, L: lockdown monthly average, PoL: post-lockdown monthly average, CI: confidence interval
