# Supplementary material for: Costs of hospital stays in Switzerland during the COVID-19 pandemic: a comparative analysis between cancer and non-cancer patients
Source: BMC Health Serv Res. 2026 Apr 24;26:934. doi: 10.1186/s12913-026-14585-0 (PMC13343702; doi:10.1186/s12913-026-14585-0)
Supplement: Supplementary file 3 — Supplementary Material 3 [file 12913_2026_14585_MOESM3_ESM.docx]

**Appendix 3, methodological aspects**

***Difference in differences models***

The model used to analyze the natural logarithm of the hospitalization cost is a linear model that requires a normal distribution of the outcome. This transformation is justified as it makes the distribution more consistent with a normal distribution [29].

Linear regression with a difference-in-differences (DiD) design:

$$Ln=\beta_{0}+\beta_{1}Cancer_{i}+\beta_{2}Lock_{i}+\beta_{3}\left( Cancer_{i}\cdot Lock_{i} \right)+\beta_{4}PostLock_{i}+\beta_{5}\left( Cancer_{i}\cdot PostLock_{i} \right)+{X'}_{i}\theta+ \varepsilon_{i}$$

Where:

- $\ln(Y_{i})$is the natural logarithm of the cost of stay i
- $Lock_{i}$ is a binary variable equal to 1 if the individual i’s stay began started during the lockdown;
- $PostLock_{i}$ is a binary variable equal to 1 if the individual i’s stay began started during the post-lockdown;
- $\beta_{0}$ is the intercept of the regression;
- $\beta_{1}$ is the baseline difference in outcome between cancer and non-cancer groups;
- $\beta_{2}$is the lockdown difference in outcome level for the non-cancer group;
- $\beta_{3}$is the difference in outcome difference between cancer and non-cancer groups during the lockdown;
- $\beta_{4}$is the post-lockdown difference in outcome for the non-cancer group;
- $\beta_{5}$is the difference in outcome difference between cancer and non-cancer groups during the post-lockdown;
- $\theta$ is the vector of the coefficients of the regression associated with other control variables (${X'}_{i}$);

The control variables ${X'}_{i}$, defined in detail in Appendix 2, are:

- Time
- Comorbidities
- In-hospital death
- University hospital
- Length of stay (LOS)
- Male
- Age fixed effect (baseline 40 years old)
- Canton fixed effect (baseline: ZH)
- 12-Month indicator

In the results, the table contains the exponentiated coefficients, $e^{\beta}$. The confidence level is set to p<0.05.

***Robust regression, estimation procedure***

This method first applies Huber weight to reduce the influence of outlying observations by progressively limiting their weight through iterative reweighting. Subsequently, a Tukey biweight estimator further refines the fit by assigning zero weight to observations with large residuals (extreme values) [52].

***Results***

**Table:** Results of the difference-in-differences analysis on the natural logarithm of the cost of stay, physician services cost, imaging cost, emergency cost, ICU cost and operation room cost, comparing the lockdown period with the pre-lockdown period between cancer and non-cancer patients.

|  | Cost of stay | | ES cost of stay | | ICU cost of stay | | OR cost of stay | | Physician cost of stay | | Nurse cost of stay | | Imaging cost of stay | |
| --- | --- | --- | --- | --- | --- | --- | --- | --- | --- | --- | --- | --- | --- | --- |
|  | Coef | 95% CI | Coef | 95% CI | Coef | 95% CI | Coef | 95% CI | Coef | 95% CI | Coef | 95% CI | Coef | 95% CI |
| Lockdown | 1.117*** | [1.113; 1.120] | 1.130*** | [1.124; 1.136] | 1.184*** | [1.155; 1.213] | 1.029*** | [1.022; 1.036] | 1.094*** | [1.090; 1.098] | 1.134*** | [1.130; 1.138] | 1.198*** | [1.188; 1.208] |
| Cancer | 1.106*** | [1.103; 1.108] | 0.998 | [0.994; 1.002] | 0.923*** | [0.909; 0.937] | 1.137*** | [1.132; 1.143] | 1.095*** | [1.092; 1.098] | 1.134*** | (1.132 - 1.137) | 1.130*** | [1.124; 1.137] |
| **Lockdown x Cancer** | **1.049***** | **[1.040; 1.058]** | **0.998** | **[0.984; 1.013]** | **0.791***** | **[0.748; 0.837]** | **1.049***** | **[1.031; 1.067]** | **1.022***** | **[1.012; 1.033]** | **1.026***** | **(1.017 - 1.035)** | **1.007** | **[0.987; 1.027]** |
| Post-lockdown | 1.099*** | [1.096; 1.101] | 1.099*** | [1.095; 1.103] | 1.100*** | [1.081; 1.118] | 1.042*** | [1.037; 1.047] | 1.066*** | [1.063; 1.069] | 1.094*** | [1.091; 1.096] | 1.141*** | [1.135; 1.148] |
| Post-lockdown x Cancer | 1.038*** | [1.034; 1.043] | 0.998 | [0.991; 1.006] | 0.865*** | [0.842; 0.890] | 1.007* | [0.999; 1.016] | 1.026*** | [1.021; 1.032] | 1.020*** | (1.016 - 1.025) | 1.010** | [1.000; 1.020] |
| Time | 0.998*** | [0.998; 0.998] | 1.001*** | [1.001; 1.001] | 1.006*** | [1.006; 1.006] | 1.002*** | [1.001; 1.002] | 0.998*** | [0.998; 0.998] | 0.998*** | (0.998 - 0.998) | 0.998*** | [0.998; 0.999] |
| Comorbidities >= 1 | 1.094*** | [1.093; 1.096] | 1.067*** | [1.065; 1.070] | 1.290*** | [1.279; 1.302] | 0.917*** | [0.915; 0.920] | 1.130*** | [1.128; 1.132] | 1.138*** | (1.137 - 1.140) | 1.150*** | [1.147; 1.154] |
| In-hospital death | 1.160*** | [1.156; 1.165] | 1.072*** | [1.067; 1.078] | 2.766*** | [2.724; 2.810] | 0.497*** | [0.492; 0.501] | 0.935*** | [0.930; 0.939] | 1.256*** | [1.251; 1.262] | 1.366*** | [1.355; 1.377] |
| University hospital | 1.239*** | [1.237; 1.242] | 2.324*** | [2.316; 2.332] | 1.753*** | [1.729; 1.777] | 1.282*** | [1.278; 1.287] | 0.924*** | [0.922; 0.926] | 1.137*** | (1.135 - 1.139) | 1.355*** | [1.349; 1.362] |
| LOS | 1.070*** | [1.070; 1.070] | 1.003*** | [1.003; 1.003] | 1.054*** | [1.054; 1.054] | 1.044*** | [1.044; 1.044] | 1.051*** | [1.051; 1.052] | 1.113*** | (1.113 - 1.113) | 1.036*** | [1.035; 1.036] |
| Male | 1.016*** | [1.014; 1.017] | 0.970*** | [0.969; 0.972] | 0.876*** | [0.868; 0.883] | 0.926*** | [0.924; 0.928] | 1.031*** | [1.030; 1.033] | 1.094*** | (1.092 - 1.095) | 0.966*** | [0.963; 0.969] |
| Age fixed effect | YES | | YES | | YES | | YES | | YES | | YES | | YES | |
| Canton fixed effect | YES | | YES | | YES | | YES | | YES | | YES | | YES | |
| 12-month correlation | YES | | YES | | YES | | YES | | YES | | YES | | YES | |
| Constant | 20'184*** | [19'313; 21'094] | 206.9*** | [192.5; 222.4] | 24.99*** | [18.31; 34.10] | 826.5*** | [758.3; 900.8] | 2'672*** | [2'534; 2'818] | 2'725*** | [2'608; 2'847] | 646.8*** | [579.6; 721.8] |
| Observations | 3'944'239 | | 1'793'259 | | 330'081 | | 1'970'193 | | 3'841'215 | | 3'764'270 | | 1'960'331 | |
| R-squared | 0.712 | | 0.204 | | 0.641 | | 0.224 | | 0.514 | | 0.872 | | 0.165 | |

Confidence level: *** p<0.01, ** p<0.05, * p<0.1; Coef: coefficient; CI 95%: 95% confidence interval; ES: Emergency service; ICU: Intensive Care Unit; OR: Operation Room; LOS: Length Of Stay

***Parallel trend assumption***

The following figures illustrate the cost trends between January 2019 and February 2019 to visually assess the parallel trends assumption between cancer and non-cancer patients’ costs.


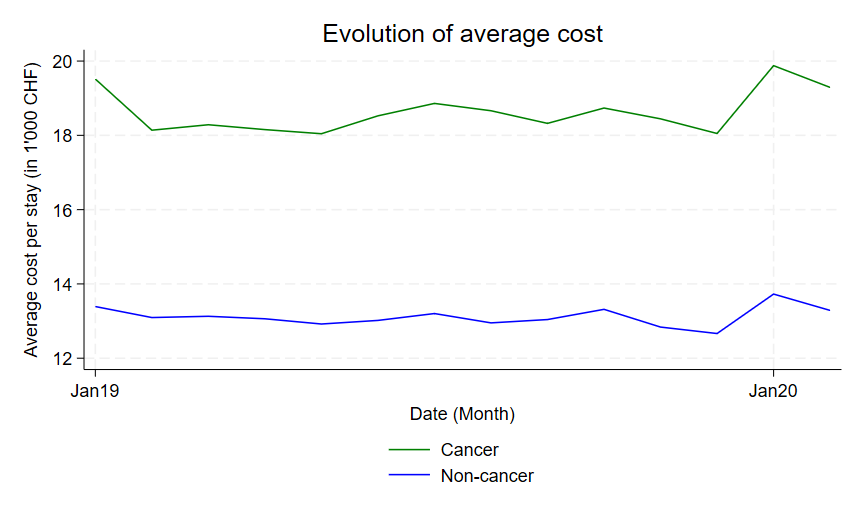

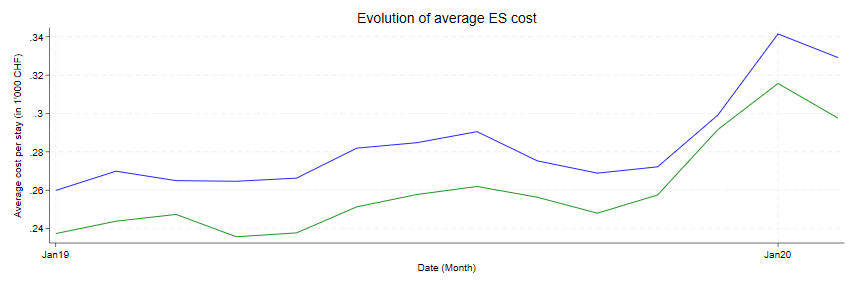

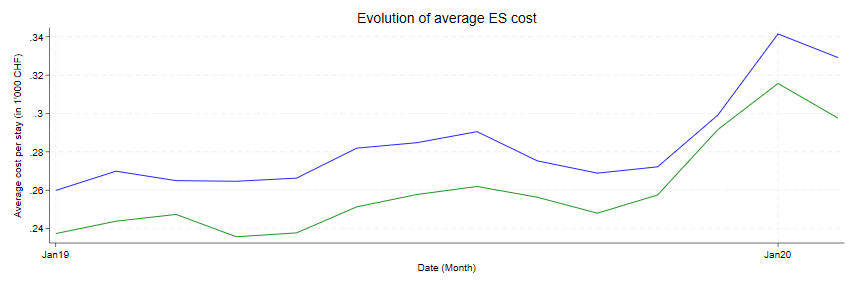

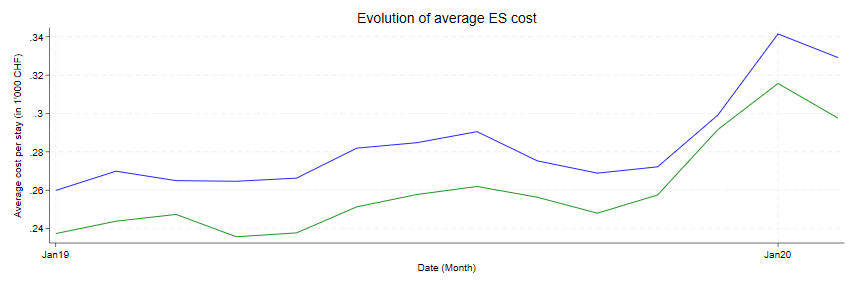

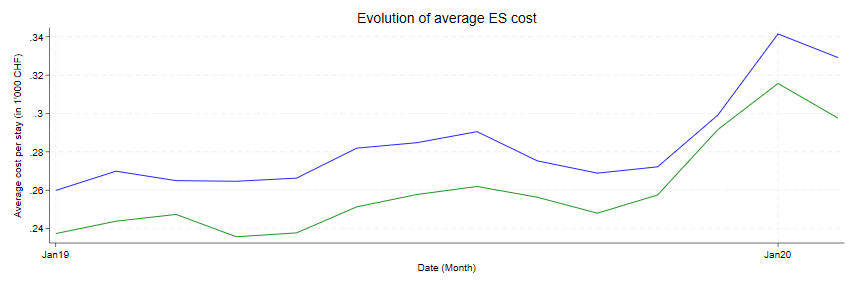

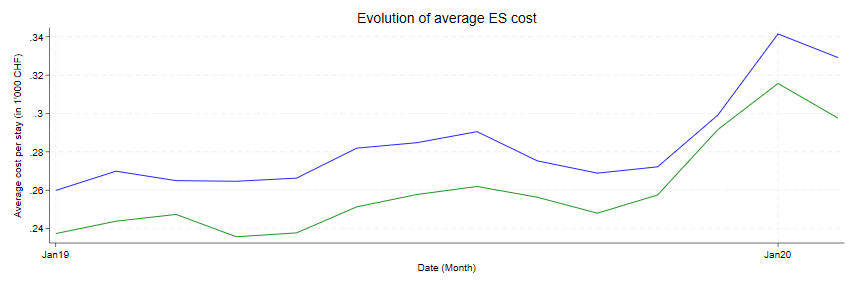

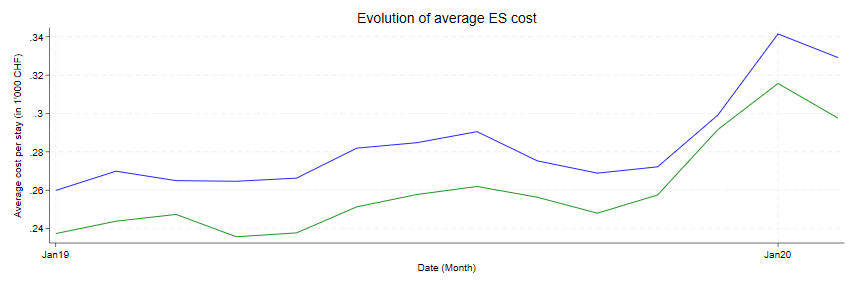


**Figure**: Trends in the average costs per stay (in 1,000 CHF), and daily average costs per stay, for cancer and non-cancer patients in Switzerland between January 2019 and February 2020.

***Marginal effects calculation***

Non-linear models present challenges in interpreting coefficients compared to linear models. For this reason, the analysis yields Average Marginal Effects (AME), providing a solution by allowing interpretation at the population level while considering the full parameter distribution. The marginal effect for each observation was calculated using its observed values for all covariates and then averaged across the sample:

$$\frac{1}{N}\cdot\sum_{i=1}^{N} \frac{\delta E(y_{i}|{Cancer_{i}}_{i}, x_{i})}{\delta Cancer}\cdot\beta_{k}$$

Where:

- N is the total number of stays
- y is the cost outcome of interest
- $x$ are the controls of the model
- $\beta_{k}$ are the parameters of interest
- $Cancer$ is the binary variable indicating having cancer as diagnosis or not

The AME are calculated at specific values to investigate the effect of the cancer binary on the outcomes in different scenarios:

1. During the pre-lockdown period (lockdown = 0 and post-lockdown = 0)
2. During the lockdown period (lockdown = 1 and post-lockdown = 0)
3. During the post-lockdown period (lockdown = 0 and post-lockdown = 1)
